# The anaerobic linalool metabolism in *Thauera linaloolentis* 47 Lol

**DOI:** 10.1186/s12866-016-0693-8

**Published:** 2016-04-27

**Authors:** Robert Marmulla, Edinson Puentes Cala, Stephanie Markert, Thomas Schweder, Jens Harder

**Affiliations:** Department of Microbiology, Max Planck Institute for Marine Microbiology, Celsiusstr. 1, D-28359 Bremen, Germany; Department of Pharmaceutical Biotechnology, Institute for Pharmacy, University of Greifswald, Felix-Hausdorff-Str. 3, D-17487 Greifswald, Germany

**Keywords:** Monoterpene, Linalool, Geraniol, Acyclic terpene utilization

## Abstract

**Background:**

The betaproteobacterium *Thauera linaloolentis* 47Lol^T^ was isolated on the tertiary monoterpene alcohol (*R,S*)-linalool as sole carbon and energy source under denitrifying conditions. Growth experiments indicated the formation of geraniol and geranial. Thus, a 3,1-hydroxyl-Δ^1^-Δ^2^-mutase (linalool isomerase) activity may initiate the degradation, followed by enzymes of the acyclic terpene utilization (Atu) and leucine/isovalerate utilization (Liu) pathways that were extensively studied in *Pseudomonas* spp. growing on citronellol or geraniol.

**Results:**

A transposon mutagenesis yielded 39 transconjugants that could not grow anaerobically on linalool and nitrate in liquid medium. The deficiencies were apparently based on gene functions required to overcome the toxicity of linalool, but not due to inactivation of genes in the degradation pathway. Growing cultures formed geraniol and geranial transiently, but also geranic acid. Analysis of expressed proteins detected several enzymes of the Atu and Liu pathways. The draft genome of *T. linaloolentis* 47Lol^T^ had *atu* and *liu* genes with homology to those of *Pseudomonas* spp..

**Conclusion:**

The in comparison to monoterpenes larger toxicity of monoterpene alcohols is defeated by several modifications of the cellular structure and metabolism in *Thauera linaloolentis* 47Lol^T^. The acyclic terpene utilization pathway is used in *T. linaloolentis* 47Lol^T^ during growth on (*R,S*)-linalool and nitrate under anoxic conditions. This is the first experimental verification of an active Atu pathway outside of the genus *Pseudomonas*.

**Electronic supplementary material:**

The online version of this article (doi:10.1186/s12866-016-0693-8) contains supplementary material, which is available to authorized users.

## Background

**Fig. 1:**
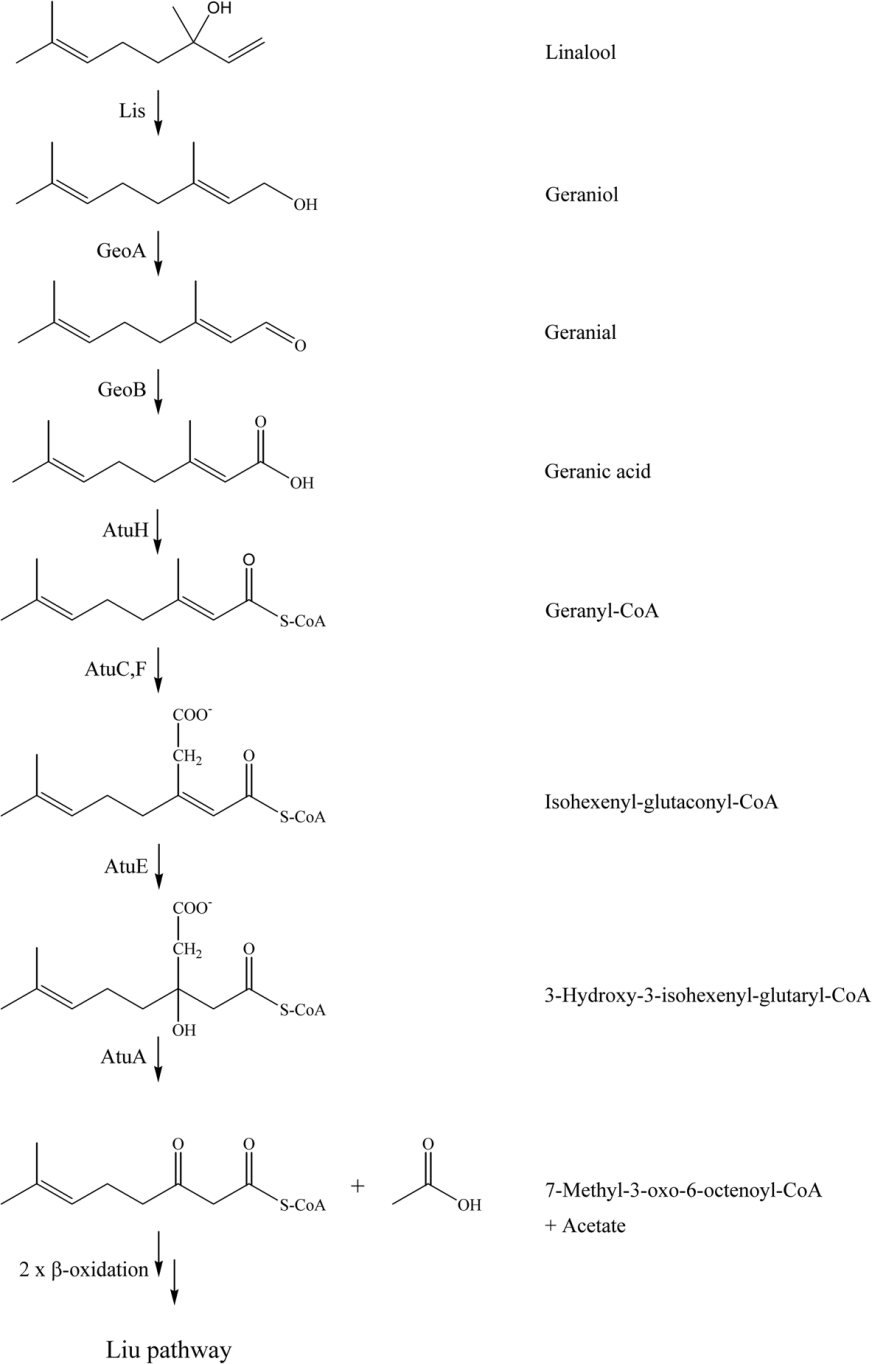
Proposed pathway for the linalool metabolism in *T. linaloolentis* 47Lol^T^, based on the acyclic terpene utilization pathway (Atu) in *Pseudomonas* spp. (adapted from [9]). Linalool isomerase (Lis), geraniol dehydrogenase (GeoA), geranial dehydrogenase (GeoB)

Linalool (C_10_H_18_O, 3,7-dimethylocta-1,6-dien-3-ol), a tertiary monoterpene alcohol, is the main constituent in essential oils of lavender and coriander. It is also a fragrance of flowers [1, 2]. As tertiary alcohols cannot be directly oxidized to ketones, microorganisms initiate their metabolism with oxidation reactions by oxygenases at other parts of the molecule [3] or with isomerizations without molecular oxygen. The latter case has been described for the betaproteobacterium *Castellaniella defragrans* 65Phen. A linalool dehydratase/isomerase metabolizes (*S*)-linalool to geraniol [4, 5]. The further degradation pathway to geranic acid is catalyzed by a NAD^+^-dependent geraniol and a geranial dehydrogenase (GeoA, GeoB) [6]. However, a pathway for geranic acid degradation was not found in the genome of *C. defragrans* 65Phen [7]. Expected were genes and operons as described for the degradation of the monoterpene alcohols geraniol and citronellol in *Pseudomonas aeruginosa* PAO1. In this strain, oxidation of the alcohols to their corresponding carboxylic acids and the oxidation of citronellic acid to geranic acid is followed by activation as CoA thioester (Fig. 1). The tertiary carbon atom is transformed into a secondary carbon atom by carboxylation of the β-methyl group as initial reaction. Hydration on the C3 atom yields 3-hydroxy-3-isohexenylglutaryl-CoA followed by acetate cleavage which removes the methyl group initially present at the tertiary carbon atom. 7-methyl-3-oxo-6-octenoyl-CoA is the product of the pathway that was named acyclic terpene utilization (Atu). For several *Pseudomonas* species the enzymes were characterized or tentatively identified: citronellol/citronellal dehydrogenase (AtuB and AtuG), citronellyl-CoA dehydrogenase (AtuD), long-chain acyl-CoA synthase (AtuH), geranyl-CoA carboxylase (AtuC and AtuF), isohexenyl-glutaconyl-CoA hydratase (AtuE) and 3-hydroxy-3-isohexenylglutaryl-CoA:acetate lyase (AtuA). The corresponding genes are arranged in an operon like structure (*atuABCDEFGH*) under the control of a transcriptional regulator (AtuR) [8–11]. 7-methyl-3-oxo-6-octenoyl-CoA undergoes two rounds of β-oxidation to two acetyl-CoA and 3-methyl-crotonyl-CoA. The latter enters the leucine/isovalerate utilization pathway (Liu) for complete mineralization [12].

The absence of the Atu pathway in the denitrifying *C. defragrans* 65Phen motivated us to search for the pathway in another denitrifying betaproteobacterium, *T. linaloolentis* 47Lol^T^. It was isolated on linalool as sole carbon and energy source under denitrifying conditions and uses linalool and geraniol as growth substrates. It has a strictly respiratory metabolism, using molecular oxygen, nitrate, nitrite or nitrous oxide as terminal electron acceptor [13]. Growth studies revealed the regioselective formation of geraniol from linalool in the stationary phase, while linalool and geranial were detected in geraniol-grown cultures. These findings suggested the presence of a linalool isomerase, a 3,1-hydroxyl-Δ^1^-Δ^2^-mutase [14]. In this study, we attempted the identification of genes and proteins essential for the anaerobic linalool degradation by transposon mutagenesis and by mass spectrometry analyses of proteins.

## Results and discussion

### Transposon insertion mutagenesis

Five thousand nine hundred sixteen transconjugants were screened on solid medium for denitrifying growth on acetate or linalool as carbon source, presenting a gene coverage of 74 % for an average gene size of 1000 bp. 92 mutants had a growth deficiency on linalool, thus a mutation frequency of 0.0155 was observed. Transfer cultures in liquid medium on linalool and nitrate revealed for 53 of these transposon mutants a biomass yield equal to the wildtype, only 39 transconjugants lacked growth on linalool. The transposon insertion sites in the genome were identified by sequencing and comparison to the genome sequence. Surprisingly, genes of the Atu pathway were not inactivated by transposon insertions. The majority of the inactivated genes was affiliated to the following functional classifications (Additional file 1: Table S1): DNA modification/processing (10 mutants), cellular transport systems (7 mutants), membrane integrity (4 mutants), miscellaneous (7 mutants) and unclassified (11 mutants). In general, these genes, involved in biosynthesis and repair, have to be seen as reflection of the cell toxicity of monoterpenes rather than the involvement in the biodegradation. Monoterpene alcohols exhibit usually a higher toxicity than the pure hydrocarbons. Once these compounds have passed the polar part of the lipopolysaccharide layer (LPS), they can integrate into the outer and inner membranes as well as the hydrophobic core of proteins. The amphiphilic monoterpene alcohols may act as surfactants. Destabilization of the membrane system and cellular structures causes ion leakages and a collapse of the proton motive force. Effects on proteins and on enzyme activities have been reported [15–17]. As defense against the penetration and accumulation of lipophilic substances, Gram-negative bacteria alter the composition and structure of their outer membrane (OM) and the lipopolysaccharide layer [18, 19], e.g., a change in membrane composition from saturated fatty acids to cyclopropane-containing fatty acids has been observed from acetate-grown to monoterpene-grown cells of *C. defragrans* 65Phen [20]. Among the transconjugants were three mutants affected in LPS core and O-antigen synthesis or modification, while another mutant was affected in a penicillin-binding protein (PDB 2) involved in peptidoglycan synthesis during cell growth and division [21, 22]. Bacteria counteract the toxic effects of monoterpenes by active export with energy-driven transporters. Well known examples for such transport systems are multidrug resistance (MDR) or resistance-nodulation-cell division (RND) efflux pumps, however the substrate range of the exporters is often not known [23–25]. We obtained linalool-growth deficient transconjugants with insertion into an ABC-type multidrug transporter or branched-chain amino acid transporters (three mutants). Ten insertions were in genes involved in DNA modification and transcriptional control. Two transconjugants had inactivated a putative transcriptional repressor of the TetR family (NCBI:ENO85695) that had a different domain order, but still an overall 38 % identity on the amino acid level (blastp) to AtuR (NCBI:BAT64851), a specific transcriptional regulator for the acyclic terpene utilization in *Pseudomonas* spp. [26]. However, the *atu* genes in 47Lol^T^ are not arranged in the operon structure of *Pseudomonas* and are distant to the repressor gene. Insertion mutants of *P. aeruginosa* showed a consecutive basal expression of Atu proteins [26]. Thus the transcriptional role of this putative repressor remains unclear. Another transconjugant had an insertion in a transcriptional regulator of the ModE family, known to be involved in molybdenum uptake and incorporation [27]. The oxidation of geraniol to geranic acid in *P. aeruginosa* PAO1 is dependent on molybdenum, but other *Pseudomonas* species grow on geraniol and express the Atu pathway without evidence for a molybdenum requirement [10].

The high frequency of transconjugants with a deficiency in growth on linalool coincides with related studies that revealed distinct changes in the transcriptome and proteome in cells switching the growth substrate from non-toxic aliphatic short chain acids to monoterpenes. *Pseudomonas* sp. M1 changed the expression of nearly 30 % of the genome in response to a change from lactate to myrcene, including genes involved in the Atu-pathway, citric acid cycle and β-oxidation, genes for a restructuring of the LPS layer and membranes and an up-regulation of nitrate-respiration gene clusters [28]. The toxicity of monoterpenes was also reflected in the proteomes of *C. defragrans* 65Phen cells grown on acetate and α-phellandrene: among the 107 identified induced proteins were several transporters and membrane biosynthesis proteins [7]. For this organism, a similar transposon mutagenesis identified the central degradation pathway for monoterpenes [7], but these alkenes are less toxic than monoterpene alcohols [6].

The lack of mutants in the catabolic pathway may also indicate the presence of isoenzymes or a second pathway for the degradation of linalool. As second pathway, a linalool kinase/monoterpene synthase together with a degradation of cyclic monoterpenes may be considered for anoxic conditions. We did not detect isoenzymes of the acyclic terpene utilization (Atu) and leucin isovalerate utilization (Liu) pathway (see Table 1). Candidate genes for a second pathway were also not found: monoterpene synthases or the *ctm* operon that was recently identified for limonene degradation [7]. The cytochrome P450 enzyme linalool 8-monooxygenase was also not present, albeit the genome contains mono- and dioxygenases. We tested growth of the transposon mutants on linalool only under denitrifying conditions, thus the linalool metabolism in the presence of oxygen will be studied in the future.

**Table 1:** Genes involved in the acyclic terpene utilization (Atu) and leucin isovalerate utilization (Liu) pathway

Enzyme	NCBI accession	Length [AA]	AA similarity [%]	E-value	Protein identification by MS
Linalool isomerase^a^	ENO87364	644	20	3E-10	Yes
Geraniol dehydrogenase^a^ (GeoA)	ENO84122	366	46	7E-96	Yes
Geranial dehydrogenase^a^ (GeoB)	ENO84123	456	31	7E-39	No
Acyl-CoA synthase^b^ (AtuH)	ENO87356	545	24	1E-15	No
Geranyl-CoA carboxylase^b^ beta-subunit (AtuC)	ENO87361	545	54	0	Yes
Geranyl-CoA carboxylase^b^ alpha subunit (AtuF)	ENO87362	705	52	0	Yes
Isohexenyl-glutaconyl-CoA hydratase^b^ (AtuE)	ENO87363	258	30	7E-27	No
3-Hydroxy-3-isohexenylglutaryl-CoA:acetate lyase^b^ (AtuA)	ENO84124	615	52	0	Yes
3-Methylcrotonyl-CoA carboxylase^b^ beta-subunit (LiuB)	ENO88226	535	72	0	Yes
3-Methylcrotonyl-CoA carboxylase^b^ alpha subunit (LiuD)	ENO88223	668	53	0	Yes
3-Methylglutaconyl-CoA hydratase^b^ (LiuC)	ENO88225	265	44	3E-73	No
Hydroxymethylglutaryl-CoA lyase^b^ (LiuE)	ENO88221	312	63	2E-129	No

Genes were identified by amino acids alignments using *C. defragrans* 65Phen^a^ and *P. aeruginosa* PAO1^b^ sequences as references. Proteins identified in the proteomic approach were analyzed by SDS-PAGE coupled to MALDI-ToF MS (Additional file 2: Figure S1)

### Characterization of growth on linalool

**Fig. 2:**
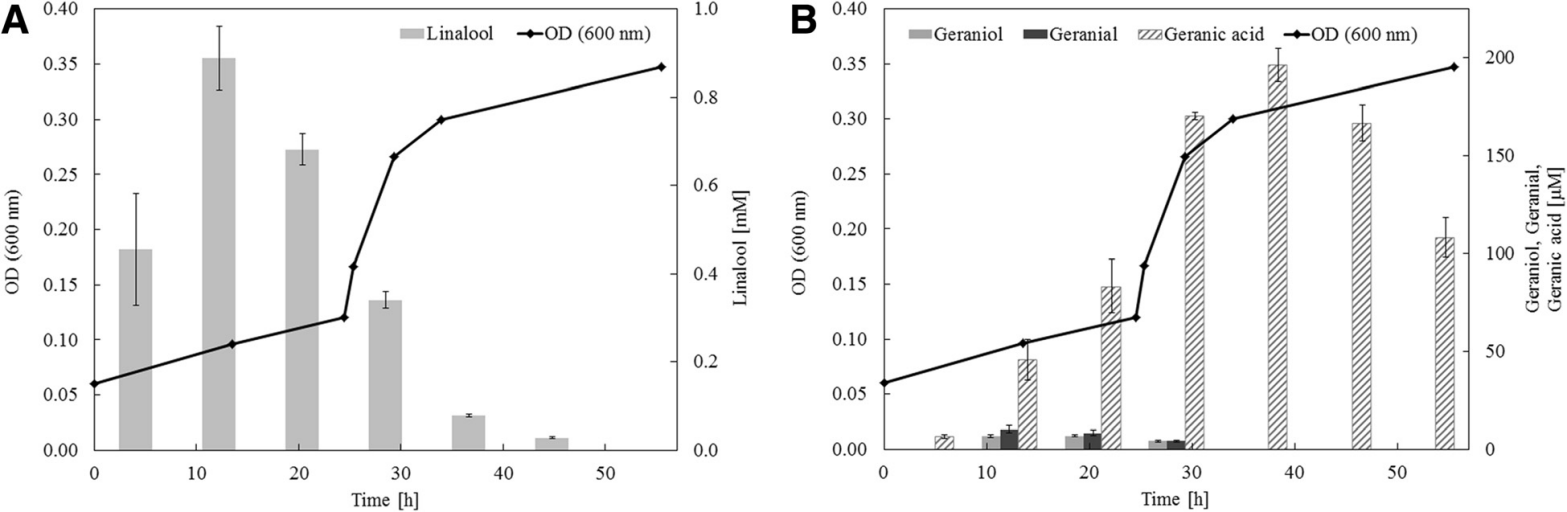
Growth of *T. linaloolentis* 47Lol^T^ on 2 mM (*R*,*S*)-linalool under denitrifying conditions. **a** Optical density at 600 nm as a measure for growth and linalool concentration in mM, **b** Optical density at 600 nm as a measure for growth and geraniol, geranial and geranic acid concentration in μM. Experiment was performed in duplicates. Initial increase in linalool concentration is due to time-dependent dissolution of linalool in medium. Linalool decreased accompanied with an increase in optical density. Geraniol and geranial accumulated only transiently, while geranic acid accumulated up to 200 μM

*T. linaloolentis* 47Lol^T^ was grown on linalool under denitrifying conditions. Besides the previously demonstrated formation of geraniol and geranial as metabolites [14] we established the detection of geranic acid (Fig. 2a,b). Geraniol and geranial accumulated transiently to 7 and 10 μM, while geranic acid accumulated up to 200 μM. Low cellular concentrations of the toxic geraniol and geranial are due to high affinities of the corresponding enzymes, which is part of a cellular defense [29]. Activities of the intial enzymes - linalool isomerase, geraniol dehydrogenase (GeoA) and geranial dehydrogenase (GeoB) - were measured in cell-free protein extracts of linalool-grown cultures. Linalool isomerase was measured for the thermodynamically favored backward reaction from geraniol to linalool and showed an activity of 260 pkat * mg protein^−1^. NAD^+^-reductase activity for geraniol and citral (mixture of geranial and neral) were 269 ± 51 pkat * mg protein^−1^ and 247 ± 107 pkat * mg protein^−1^, respectively. Abundant proteins in the cell-free protein extract were identified by SDS-PAGE and MALDI-ToF-based mass spectrometric analysis (Additional file 2: Figure S1). Proteins annotated as linalool isomerase, geraniol dehydrogenase, the two subunits of geranyl-CoA carboxylase as well as a 3-hydroxy-3-isohexenylglutaryl-CoA:acetate lyase were identified together with the two subunits of the methylcrotonyl-CoA carboxylase and the isovaleryl-CoA dehydrogenase of the leucine degradation pathway (*liu* operon).

### Genome annotation

Two publicly available draft genomes of *T. linaloolentis* 47Lol^T^ were assembled and yielded a draft genome with 23 contigs of 4,402,076 bp, an overall G + C content of 66.6 %, 4084 open reading frames, 46 transfer RNA genes and 1 ribosomal RNA operon. The genome completely covers central metabolic pathways, e.g. citric acid cycle, β-oxidation of fatty acids, oxygen respiration and denitrification. Genetic information from *P. citronellolis*, *P. aeruginosa* PAO1 (Atu and Liu pathway) and *C. defragrans* 65Phen (linalool isomerization and geraniol/geranial oxidation) were used to identify homologous genes in *T. linaloolentis* 47Lol^T^ (Table 1).

**Fig. 3:**
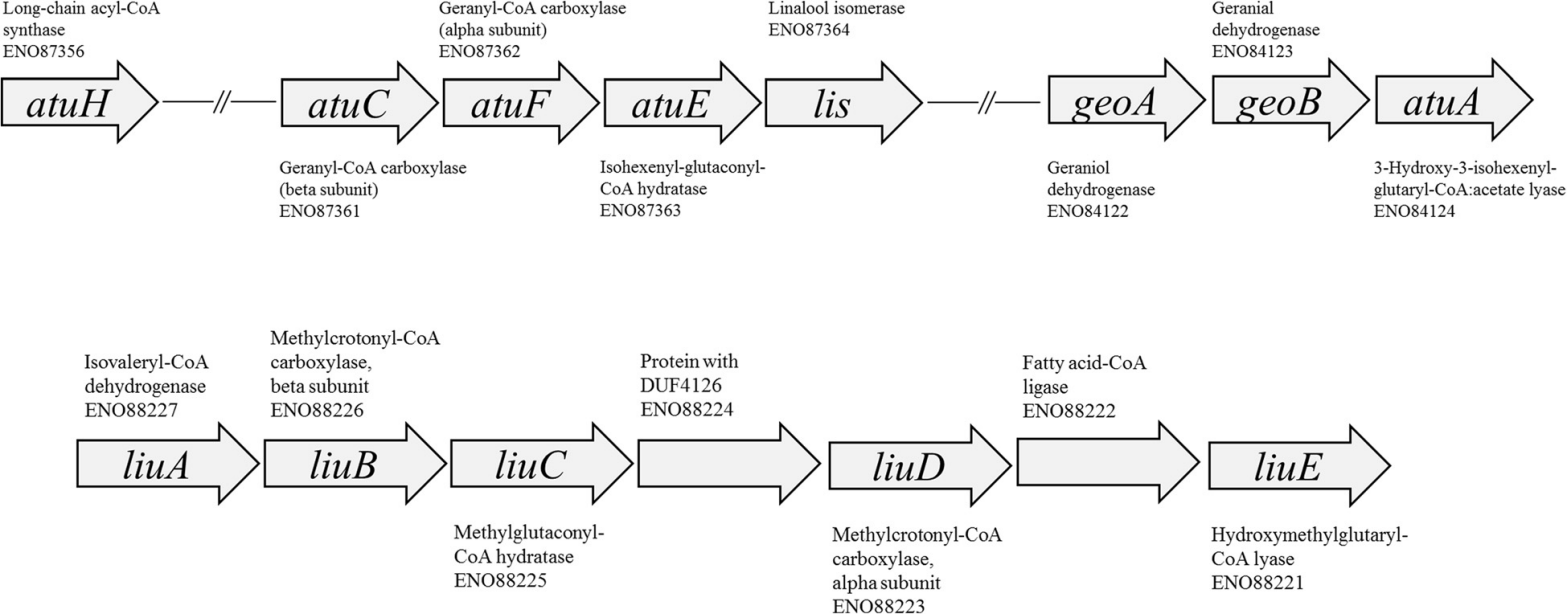
Gene arrangement of acyclic terpene utilization (*atu*) and leucine isovalerate utilization (*liu*) pathway genes in the draft genome of *T. linaloolentis* 47Lol^T^. Annotation and NCBI accession numbers are indicated. Gaps size between *atuH* and *atuC* is 3488 bp and gap size between *lis* and *geoA* is 7617 bp. The *atu* genes are located on a 388748 bp large contig, while the *liu* genes are located on a 205711 bp contig

Annotations were fully supported by the aforementioned protein identification. One result of the reassembly was the formation of two large contigs that contained all genes of the Atu and Liu pathways over a region of 24 and 9.1 kb, respectively. The *atu* genes were organized with the putative linalool isomerase gene *lis* downstream of *atuCFE* and the genes for the geraniol oxidation *geoAB* upstream of *atuA* (Fig. 3). The genes in between were annotated as hypothetical proteins. Gene homologs for *atuB* and *atuG*, the putative citronellol and citronellal dehydrogenases, were identified in the draft genome but did not show significant similarity in a reciprocal best match analysis.

## Conclusion

A transposon mutagenesis targeting the degradation pathway for linalool yielded mutants defective in the defense against the toxic monoterpene alcohol. Enzyme activities of a linalool isomerase, geraniol and geranial dehydrogenases together with the formation of geranic acid demonstrated the initial degradation pathway. Mass spectrometry identified the carboxylases of Atu and Liu pathways which indicated the utilization of these pathways for geranic acid degradation. The draft genome contained the *atu* genes together with candidate genes for the initial degradation pathway. To our knowledge, this is the first description of an active Atu pathway outside of the genus *Pseudomonas* and under anoxic conditions.

## Methods

### Bacterial strains and cultivation conditions

*T. linaloolentis* 47Lol^T^ (= CCUG 41526^T^ = CIP 105981^T^ = DSM 12138^T^ = IAM 15112^T^ = JCM 21573^T^ = NBRC 102519^T^) was isolated and maintained in our laboratory [13]. For this study, the strain was cultivated under anaerobic, denitrifying conditions in artificial fresh water (AFW) medium. Medium was prepared as described by Foss et al. [20] with modifications. Carbonate buffer was replaced by 10 mM Na_2_HPO_4_/NaH_2_PO_4_ and vitamins were omitted. The headspace contained nitrogen gas only. 20 mM Acetate or 2 mM (*R,S*)-linalool were used as growth substrates. Growth experiments were performed in 10 mL cultures, incubated at 28 °C under mild shaking (90 rpm). Growth occurred homogenous and was estimated by measuring the optical density at 600 nm. Monoterpenes were purchased from Sigma-Aldrich (Taufkirchen, Germany) and were of 95 to 97 % purity. Geranial was provided as citral; the mixture of geranial and neral.

Solid AFW medium (0.5 g L^−1^ KH_2_PO_4_, 0.5 g L^−1^ NH_4_Cl, 0.5 g L^−1^ MgSO_4_ x 7 H_2_O, 0.1 g L^−1^ CaCl_2_ x 2 H_2_O, 0.85 g L^−1^ NaNO_3_, 11.9 g L^−1^ HEPES, 15 g L^−1^ agar, pH 7.2) was mixed, autoclaved and supplemented with trace-element and selenite-tungstate stock solutions as described [20]. Plates contained either 2 mM (*R,S*)-linalool or 50 mM acetate as carbon source. Liquid brain heart infusion (BHI) medium (12 g L^−1^ brain heart infusion, 10 g L^−1^ peptone, 4 g L^−1^ hydrolyzed casein, 5 g L^−1^ NaCl, 2 g L^−1^ glucose, 2.5 g L^−1^ Na_2_HPO_4_, 0.2 g L^−1^ NaNO_3_, pH 7.4) was used to yield higher biomass for genomic DNA extraction. Antibiotics were provided from stock solutions in the following working concentrations: 25-50 μg mL^−1^ kanamycin, 50 μg mL^−1^ rifampicin, 1 μg mL^−1^ ofloxacin.

### Transposon insertion mutagenesis

Transposon insertion mutagenesis was performed by using the mini-Tn5 plasmid and biparental conjugation as described by Larsen et al. [30]. Two spontaneous *T. linaloolentis* 47Lol^T^ mutants resistant to the antibiotics ofloxacin (47Lol-OF) or rifampicin (47Lol-RIF) were obtained by repeated culturing of the wildtype in anoxic medium on 20 mM acetate and 10 mM nitrate in the presence of various concentrations of the antibiotics (20 – 200 μg mL^−1^ rifampicin, 0.05 – 0.15 μg mL^−1^ ofloxacin). *Escherichia coli* BW20767 [31] harboring the pRL27 plasmid with the mini-Tn5 transposon was grown overnight in lysogeny broth with 50 μg mL^−1^ kanamycin at 37 °C. Cells were collected by centrifugation (8000 x *g*, 5 min) and washed twice with fresh medium without antibiotic. The cells were resuspended in medium, diluted to an optical density (600 nm) of 1 and mixed in a donor:recipient ratio of 1:3. 200 μL of this mixture were incubated on AFW-plates (50 mM acetate, no antibiotics) at 28 °C for 24 h aerobically. Cells were resuspended in 1 mL AFW and diluted twofold and tenfold and plated on AFW-plates containing 50 mM acetate, kanamycin and rifampicin or ofloxacin. The plates were incubated for up to 5 days aerobically at 28 °C. Colonies were randomly screened for transposon-integration in the genome by PCR with the primer pair pRL27 Tn5_F (CGTTACATCCCTGGCTTGTT) and pRL27 Tn5_R (TGAAGAAGGTGTTGCTGA) [7]. Transconjugants were replica-plated on AFW-plates containing either 20 mM acetate or 2 mM (*R,S*)-linalool and nitrate. Colonies showing deficiency for anaerobic growth on linalool were selected and transferred into liquid culture and a second anoxic screening on acetate and linalool in the presence of nitrate was performed. Cultures deficient in growth on linalool were selected for further analysis. Genomic DNA was prepared to determine the location of transposon insertion and to identify the affected gene by direct sequencing. Mutants were grown in 10 mL BHI medium overnight. Biomass was collected by centrifugation (4500 x *g*, 10 min), resuspended in 150 μL buffer (Tris-Cl 50 mM, 10 mM EDTA, pH 8, 50 units RNase A) and treated with 150 μL lysis buffer (200 mM NaOH, 1 % w/v SDS). After mixing and incubation at 96 °C for 20 min the solution was neutralized by addition of 225 μL neutralization buffer (3 M sodium acetate, pH 5.5). Precipitates were removed by centrifugation (20000 × *g*, 10 min). Genomic DNA was precipitated from the supernatant by addition of ice-cold isopropanol to a content of 50 % (v/v), incubation at −20 °C for 60 min and centrifugation (20000 × *g*, 20 min, 4 °C). The DNA pellet was washed with 70 % (v/v) ice-cold ethanol, centrifuged and dissolved in 40 μL water. Sequencing reactions were performed with the BigDye Terminator v3.1 Cycle Sequencing Kit (Applied Biosystems by Thermo Fisher Scientific, Waltham, USA), gDNA (1 – 3 μg) as template and the primer pair tnpRL 17_1 (AACAAGCCAGGGATGTAA) and tnpRL 13_2 (CAGCAACACCTTCTTCACGA) [30] using the following program: 96 °C for 20 s, 99 cycles of 96 °C for 10 s, 56 °C for 5 s, 60 °C for 4 min. Amplicons were analyzed with an ABI Prism 3130*xl* Genetic Analyzer (Applied Biosystems by Thermo Fisher Scientific, Waltham, USA). The probability of coverage of a gene by a mutant was calculated for a genome size of 4402076 bases with:

$$ P=1-{\left(1-\frac{average\kern0.5em  gene\kern0.5em  size}{genome\kern0.5em  size}\right)}^{number\kern0.5em  of\kern0.5em  mutans} $$

### Metabolite analysis

*T. linaloolentis* 47Lol^T^ was cultivated on 1.5 mM (*R,S*)-linalool and 10 mM nitrate in duplicate cultures. Inoculum was 2 % (v/v) of a linalool-adapted culture. Two cultures with similar optical densities were sampled for metabolite extraction, covering growth over the lag-phase, exponential phase to the entry into the stationary phase. 10 mL culture liquid was transferred to a 15 mL plastic tube, 3 g of NaCl and 250 μL of n-hexane were added. The sample was mixed by vortexing for 30 s and 10 min incubation on a tilting shaker. Phase separation was achieved by 10 min centrifugation (3500 × *g*, 10 min, 5 °C). The clear hexane phase was recovered into a GC-vial. 1 μL sample was analyzed by gas chromatography with flame ionization detection (Shimadzu GC-14A, Shimadzu, Duisburg, Germany) on a Hydrodex-β-6TBDM column (25 m × 0.25 mm, Macherey-Nagel, Düren, Germany) with the following temperature program: injection port 200 °C, detection port 250 °C, initial column temperature 60 °C for 1 min, increasing to 130 °C at a rate of 5 °C min^−1^, keeping 130 °C for 0.5 min, followed by an increase to 230 °C at 20 °C min^−1^ and hold for 4 min. To determine the geranic acid concentration, 50 μL of the hexane phase were mixed with 50 μL 1 mM NaOH and the hexane was allowed to evaporate. 5 μL of 20 mM phosphoric acid were added and the sample was analyzed by an Acquity UPLC H-class system (Waters Corporation, Milford, USA). 1 μL sample was separated on a reverse phase (BEH C18, 1.7 μm, 2.1 × 50 mm) column with 1 mM H_3_PO_4_ at 0.6 mL min^−1^ in a water-acetonitrile gradient from 10 to 70 % acetonitrile (v/v) at 30 °C. UV detection was performed at 221 nm.

### Geraniol-, Geranial dehydrogenase and Linalool isomerase assays

Soluble protein extract of *T. linaloolentis* 47Lol^T^ was prepared from biomass suspension in Tris-Cl buffer (40 mM, pH 8.0) by passing through an One-Shot cell disruptor (Constant Systems Ltd., Daventry, UK) at 1.7 GPa two times, followed by ultracentrifugation (150000 × *g*, 30 min, 4 °C). The clarified supernatant was used for further experiments. Geraniol- and Geranial dehydrogenase activities were determined by absorption measurement of NADH formation at 340 nm on a Beckman DU640 Spectrophotometer (Beckman Coulter, Brea, USA) as described previously [6]. Soluble protein extract was dialyzed against Tris-Cl buffer (VISKING dialysis tubing, 14 kDa cutoff, Serva, Heidelberg, Germany). The final assay (1 mL) was performed in a quartz cuvette at 22 °C and contained 1 mM geraniol or 1.5 mM citral, 1 mM NAD^+^ (final concentrations) and various amounts of protein. Reactions were started by addition of NAD^+^. Rates were calculated based on a molar extinction coefficient of 6220 M^−1^ cm^−1^. Linalool isomerase activity was determined in soluble protein extracts. Glass vials (4 mL) with 300 to 500 μL sample were reduced with 5 mM dithionite, closed with butyl rubber stoppers and flushed with nitrogen gas for 3 min, to provide anoxic conditions. Samples were incubated for 20 min at room temperature. The reaction was started by addition of 200 μL geraniol (200 mM) in 2,2,4,4,6,8,8-heptamethylnonane (HMN) through a needle and incubated for 14 to 16 h at 28 °C under mild shaking. Product formation was determined in samples from the HMN phase by gas chromatography with flame ionization detection (PerkinElmer Auto System XL, Überlingen, Germany) on an Optima-5column (30 m × 0.32 mm, 0.25 μm film thickness; Macherey-Nagel, Düren, Germany) with the following temperature program: injection port 250 °C, detection port 350 °C, initial column temperature 40 °C for 2 min, increasing to 100 °C at a rate of 4 °C min^−1^, keeping 100 °C for 0.1 min, followed by an increase to 320 °C at 45 °C min^−1^ and hold for 3 min. The split ratio was set to 1:9.

### Proteomics by MALDI-ToF MS

*T. linaloolentis* 47Lol^T^ cultures were grown on (*R,S*)-linalool to the late exponential phase and harvested by centrifugation. Cells were suspended in Tris-Cl buffer (40 mM, pH 8.0) and soluble protein extract was prepared as described above. Protein samples, obtained from individual purifications, were analyzed by SDS-PAGE coupled with matrix-assisted laser desorption/ionization time of flight (MALDI-ToF) mass spectrometry (MS). Protein bands in gels were excised manually, and the Ettan Spot Handling Workstation (GE Healthcare, Freiburg, Germany) was used for trypsin digestion and embedding of the resulting peptide solutions in an α-cyano-4-hydroxycinnamic acid matrix for spotting onto MALDI targets. MALDI-ToF MS analysis was performed on an AB SCIEX TOF/TOF™ 5800 Analyzer (Sciex, Ontario, Canada) [32]. Spectra in a mass range from 900 to 3700 Da (focus 1700 Da) were recorded and analyzed by GPS Explorer™ Software Version 3.6 (build 332, Applied Biosystems by Thermo Fisher Scientific, Waltham, USA) and the Mascot search engine version 2.4.0 (Matrix Science Ltd, London, UK) using the RAST draft genome as reference.

### Draft genome and gene analysis

Two publicly available sequencing datasets for *T. linaloolentis* 47Lol^T^, ASM31020 (4.199 Mbp on 220 contigs, available since November 2012) and ASM62130 (4.214 Mbp on 46 contigs, available since April 2014), were merged using Sequencher 4.6 (Gene Codes, Ann Arbor, USA) with a minimum match percentage of 95 % and a minimum overlap of 50 bases. The resulting draft genome was uploaded to RAST for further analysis [33, 34]. Known genes from *Castellaniella defragrans* 65Phen, *Pseudomonas aeruginosa* PAO1 and *P. citronellolis*, encoding enzymes involved in the linalool and geraniol metabolism, were used to identify homologs in *T. linaloolentis* 47Lol^T^. Overall similarity of the identified genes to their homologs was determined by blastp [35].

## Ethics approval and consent to participate

### Consent for publication

Not applicable.

### Availability of data and materials

The datasets supporting the conclusions of this article are included within the article and its additional files.

## Additional files

Additional file 1: Table S1. Transposon insertion mutants. The table presents the transposon insertion mutants (name, NCBI accession number of protein, annotation). (DOCX 22 kb) Additional file 2: Figure S1. SDS-PAGEs (A, B) of cell-free protein extracts of *T. linaloolentis* 47Lol^T^ grown on 1 mM (*R,S*)-linalool and 10 mM nitrate to the late exponential phase. Indicated bands were extracted and analyzed by MALDI-ToF MS. Marker is given in kDa. (1) AtuF - geranyl-CoA carboxylase alpha subunit, (2) LiuD - methylcrotonyl-CoA carboxylase biotin-containing subunit, (3) AtuA - 3-hydroxy-3-isohexenylglutaryl-CoA:acetate lyase, (4) AtuC - geranyl-CoA carboxylase beta subunit, (5) LiuA - Isovaleryl-CoA dehydrogenase, (6) GeoA - geraniol dehydrogenase, (7) Lis - linalool isomerase, (8) LiuB - methylcrotonyl-CoA carboxylase carboxyl transfer subunit. (PNG 263 kb)

## Abbreviation

LPS: lipopolysaccharide
