# Supplementary material for: The anaerobic linalool metabolism in Thauera linaloolentis 47 Lol
Source: BMC Microbiol. 2016 Apr 27;16:76. doi: 10.1186/s12866-016-0693-8 (PMC4847356; doi:10.1186/s12866-016-0693-8)
Supplement: Additional file 1: Table S1. — Transposon insertion mutants. The table presents the transposon insertion mutants (name, NCBI accession number of protein, annotation). (DOCX 22 kb) [file 12866_2016_693_MOESM1_ESM.docx]

**Table S1:** Transposon insertion mutants.

| Mutant | Length [bp] | NCBI  accession | Protein  annotation | Probable function |
| --- | --- | --- | --- | --- |
|  |  |  | *DNA modification / transcriptional control* |  |
| 18 | 309 | ENO90120 | Integration host factor, α-subunit | Transcription regulation |
| 36 | 2124 | ENO88423 | DNA-repair protein;  Type III restriction enzyme | DNA repair  mechanism |
| 41 | 813 | ENO89557 | DNA-binding domain of ModE | Transcription regulation (molybdenum metabolism) |
| 47 | 675 | ENO85695 | Trancriptional regulator, TetR family | Transcription regulation |
| 63 | 294 | ENO87982 | XRE family transcriptional regulator | Transcription regulation (response to xenobiotics) |
| 64 | 675 | ENO85695 | Trancriptional regulator, TetR family | Transcription regulation |
| 83 | 1074 | ENO88415 | DNA sulfur modification protein DndB | DNA binding and recognition of modification sites |
| 88 | 2568 | ENO89640 | PAS/PAC sensor hybrid histidine kinase | Signaling and transcription regulation |
| 91 | 1542 | ENO89296 | ATP-dependent endonuclease family protein | DNA repair mechanism |
| 93 | 903 | ENO82884 | Integrase core domain (Transposase) | DNA mobility |
|  |  |  |  |  |
|  |  |  |  |  |
|  |  |  | *Transport* |  |
| 37 | 2433 | ENO85793 | TonB-dependent siderophore receptor (FpvA) | Transport |
| 38 | 2430 | ENO90450 | TonB-dependent siderophore receptor | Transport |
| 60 | 1332 | ENO85236 | Branched-chain amino acid transport system permease protein LivM | Branched-chain amino acid transport |
| 69 | 705 | ENO88656 | Membrane protein belonging to the AzlC superfamily | Branched-chain amino acid transport |
| 73 | 705 | ENO88656 | Membrane protein belonging to the AzlC superfamily | Branched-chain amino acid transport |
| 80 | 1140 | ENO86020 | ABC-type multidrug transprort system | Transport |
| 81 | 2475 | ENO86288 | TonB-dependent siderophore receptor | Transport |
|  |  |  |  |  |
|  |  |  |  |  |
|  |  |  |  |  |
|  |  |  |  |  |
|  |  |  | *Membrane integrity* |  |
| 21 | 1971 | ENO84346 | Penicillin-binding protein 2 | Peptidoglycan synthesis (cell elongation) |
| 52 | 963 | ENO84489 | ADP-L-glycero-D-manno-heptose-6-epimerase (EC 5.1.3.20) | Lipooligosaccharide core biosynthesis |
| 68 | 1386 | ENO89893 | UDP-phosphate galactose phosphotransferase | Lipopolysachharide O-antigen synthesis |
| 85 | 1386 | ENO89893 | UDP-phosphate galactose phosphotransferase | Lipopolysachharide O-antigen synthesis |
|  |  |  |  |  |
|  |  |  | *Miscellaneous* |  |
| 54 | 1548 | ENO84316 | ATP-dependent RNA helicase | RNA metabolism |
| 58 | 264 | ENO89266 | Lysophospholipase (EC 3.1.1.5) | Phospholipid metabolism |
| 61 | 16533 | ENO89831 | Putative large exoprotein involved in heme utilization or adhesion of ShlA/HecA/FhaA family | Similarity to hemagglutinin-like protein; involved in cell adhesion |
| 62 | 1041 | ENO84082 | Peptidase Gluzincin family (MA2) | Protein metabolism |
| 66 | 1146 | ENO84083 | Fic (Filamentation induced by cAMP) family protein | Regulatory mechanism of cell division, folate metabolism |
| 67 | 504 | ENO85121 | Putative phasin protein | Surface proteins of intracellular storage granules |
| 87 | 504 | ENO85121 | Putative phasin protein | Surface proteins of intracellular storage granules |
|  |  |  |  |  |
|  |  |  | *Unclassified* |  |
| 28 | 7296 | ENO88318* | Hypothetical protein | unknown |
| 43 | 1812 | ENO90436* | Putative ATP and DNA-binding domain | DNA metabolism |
| 46 | 1209 | ENO90599 | Membrane protein, Acyltransferase | unknown |
| 53 | 1524;  927 | ENO87300; ENO87299 | Reverse transcriptase;  Threonine dehydratase | Unknown;  Amino acid metabolism |
| 55 | 1104 | ENO86348 | Hypothetical protein | unknown |
| 70 | 525 | ENO88413 | Hypothetical protein | Motif for dnd system-associated protein 4 within DNA sulfur modification system |
| 71 | 1179 | ENO87344 | von Willebrand factor A containing CoxE domain-like protein | unknown |
| 76 ^#^ |  |  |  |  |
| 77 | 819 | ENO89891 | Similarity to labA-like proteins (NYN domain) | Unknown (might be involved in RNA core metabolism; RNA processome) |
| 79 | 1170 | ENO89611 | Hypothetical protein | Unknown (DUF2863) |
| 84 | 1104 | ENO86348 | Hypothetical protein | Unknown |

* DNA sequence identical but translation is incorrect in NCBI

^#^ no sequence was obtained from the sequencing for mutant 76
