# Supplementary figures and images for: The anaerobic linalool metabolism in Thauera linaloolentis 47 Lol
Source: BMC Microbiol. 2016 Apr 27;16:76. doi: 10.1186/s12866-016-0693-8 (PMC4847356; doi:10.1186/s12866-016-0693-8)

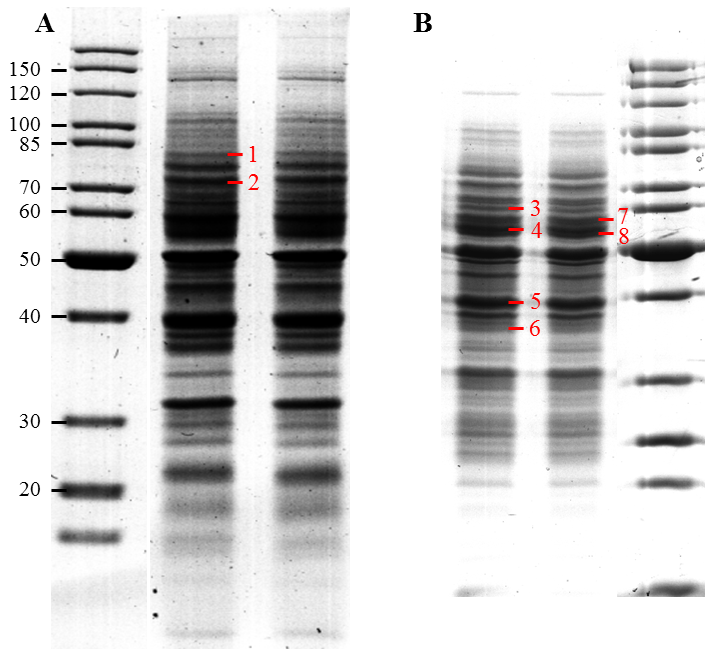

Supplement: Additional file 2: Figure S1. — SDS-PAGEs (A, B) of cell-free protein extracts of T. linaloolentis 47LolT grown on 1 mM (R,S)-linalool and 10 mM nitrate to the late exponential phase. Indicated bands were extracted and analyzed by MALDI-ToF MS. Marker is given in kDa. (1) AtuF - geranyl-CoA carboxylase alpha subunit, (2) LiuD - methylcrotonyl-CoA carboxylase biotin-containing subunit, (3) AtuA - 3-hydroxy-3-isohexenylglutaryl-CoA:acetate lyase, (4) AtuC - geranyl-CoA carboxylase beta subunit, (5) LiuA - Isovaleryl-CoA dehydrogenase, (6) GeoA - geraniol dehydrogenase, (7) Lis - linalool isomerase, (8) LiuB - methylcrotonyl-CoA carboxylase carboxyl transfer subunit. (PNG 263 kb) [file 12866_2016_693_MOESM2_ESM.png]
